# Management of a radiopaque foreign body associated with a lower first premolar: A case report

**DOI:** 10.1002/ccr3.5465

**Published:** 2022-02-17

**Authors:** Mohammed Yagmoor, Abdulaziz Bakhsh, Olfah Mandourah, Loai Alsofi

**Affiliations:** ^1^ Department of Endodontics Faculty of Dentistry King Abdulaziz University Jeddah Saudi Arabia; ^2^ Division of Endodontics Department of Restorative Dentistry Faculty of Dentistry Umm Al‐Qura University Makkah Saudi Arabia; ^3^ Bandar Al Ahmadi Dental Complex Jeddah Saudi Arabia

**Keywords:** CBCT, endodontics, root canal treatment, vertical root fracture

## Abstract

Operator mishaps significantly affects root canal treatment outcome. However, several factors affect root canal anatomy of teeth. Therefore, knowledge of teeth anatomy and the use of advanced technologies like cone‐beam computed tomography (CBCT), magnification, and illumination are crucial for better diagnostic purposes and to avoid any procedural errors. In this case report, we present a case of a 32‐year‐old male patient with a radiopaque foreign body associated with a lower first premolar tooth and how do the use of advanced technologies could affect the treatment outcome.

## Abstract 2

Knowledge of teeth anatomy and the use of advanced technologies like cone‐beam computed tomography (CBCT), magnification, and illumination are crucial for better diagnostic purposes and to avoid any procedural errors.

## 1 INTRODUCTION

The objective of root canal treatment (RCT) is to thoroughly clean and disinfect the infected root canal system to prevent further progression of infection. Therefore, a comprehensive learning of root canal anatomy as well as diagnosis is critical for successful root canal therapy.^1^ Endodontic treatment failure could be ascribed to several factors including intra‐radicular and/or extra‐radicular infections, true cyst, foreign body reaction, and cholesterol crystals.^2^, ^3^, ^4^, ^5^ Furthermore, operator mishaps could influence the outcome of root canal treatment such as broken instrument, perforation, transportation, and the extent of root canal filling.^6^

Mandibular first premolar showed high percentage of anatomical variations; this may eventually lead to catastrophic clinical errors.^7^, ^8^ It has been shown in the literature that the prevalence of mandibular first premolars exhibiting two canals was 25%.^9^ This percentage has been changed increasingly for certain populations and reached up to 39.5%, 41.8%, and 46% for Turkish, Jordanian, and Chinese populations, respectively.^10^, ^11^, ^12^ Multiple studies have demonstrated the complexity of root canal morphology. Furthermore, several predicting factors were found to influence these differences including race and ethnicity,^10^, ^12^, ^13^, ^14^, ^15^ gender,^10^ age, and study design.^16^

Root canal morphology was initially classified by Weine who introduced 4 root canal configurations^17^; this was followed by eight configurations described by Vertucci.^9^ Another two systems were introduced in 2001 and 2004, respectively.^10^, ^15^ Recently, Ahmed et al. designed a new way to classify the root and root canal morphology for those categories that were not classifiable by the previous systems.^18^

Diagnosis of root canal infection is an accumulation of clinical signs and symptoms as well as clinical and radiographic examination. Proper clinical and radiographic examination is crucial for proper diagnosis to avoid never events such as treating the wrong tooth or undergoing unnecessary treatment for the patient. Furthermore, radiographic imaging is necessary during examination as some conditions may be asymptomatic and are only detected by chance during consultation appointment including split root, impacted unerupted teeth, asymptomatic root resorption, and cemento‐osseous dysplasia.

Assessment of the root canal morphology using periapical radiograph is not feasible given its limitation as it provides a two‐dimensional image for a three‐dimensional object. Recently, the introduction of cone‐beam computed tomography (CBCT) by Tachibana and Matsumoto in 1990,^19^ and micro‐computed tomography (Micro‐CT) scans have proven their efficacy in expanding our knowledge of the anatomical variations by giving three‐dimensional (3D) shadowgraphs for better qualitative and quantitative assessments.^19^, ^20^, ^21^, ^22^, ^23^

Identification of vertical root fracture may not always be feasible from radiographic imaging as the fracture may not be wide enough for the radiation beam to pass through it. On the contrary, presence of a radiopaque foreign body could be interpreted as a split tooth or a supernumerary tooth. Therefore, dental practitioners should always seek consultation when in doubt to provide the best practice to patients.

Therefore, this case report aims to elaborate the importance of diagnosing and managing multiple mishaps in a complicated mandibular first premolar through a multi‐disciplinary approach.

## 2 CASE DESCRIPTION

**FIGURE 1:**
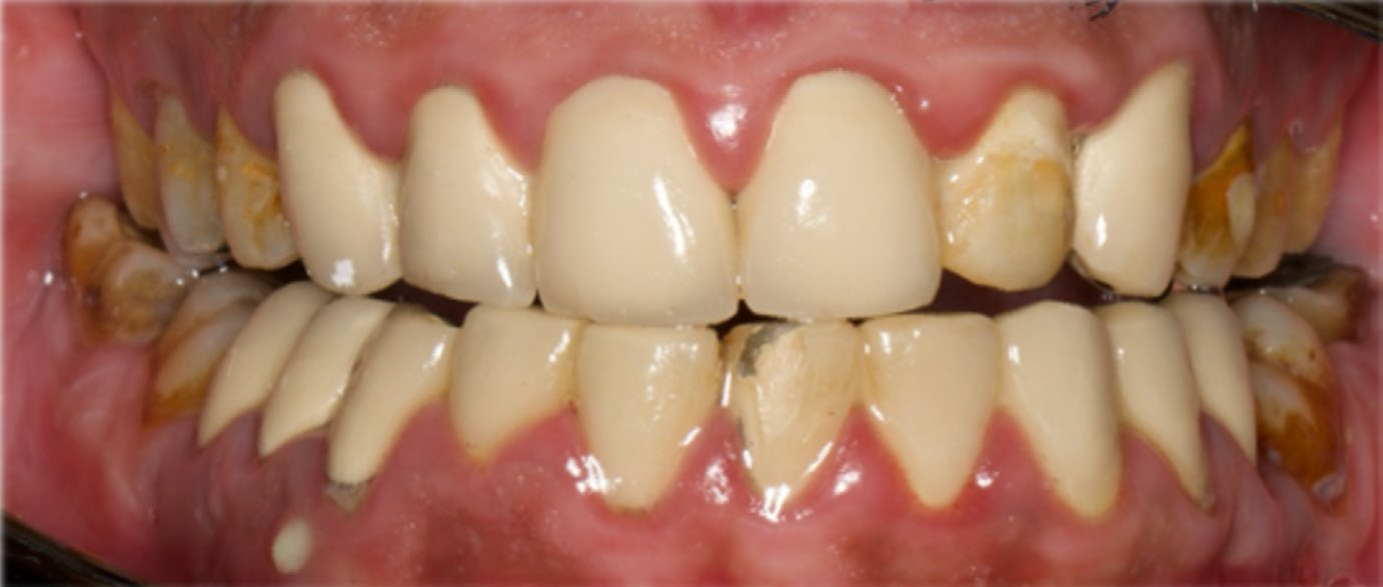
Intra‐oral image showing a discharging sinus with tooth #44

**FIGURE 2:**
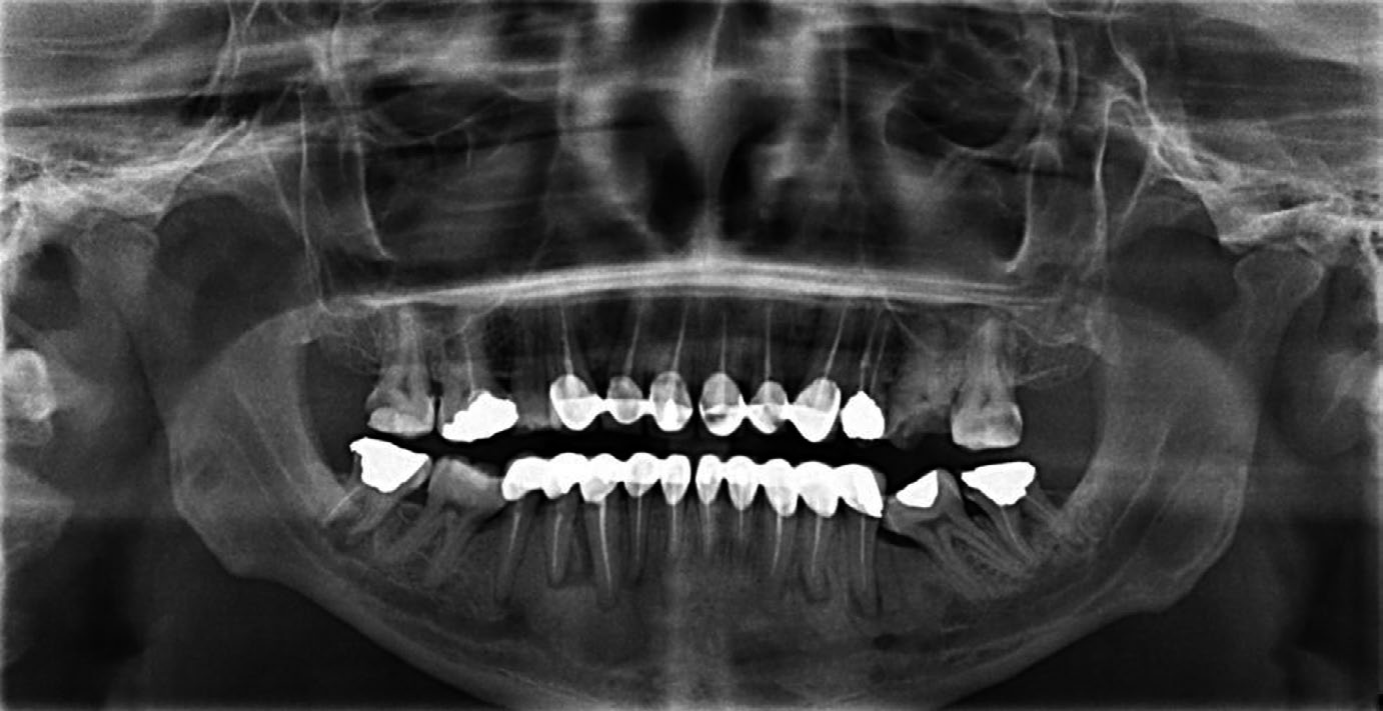
Panoramic radiograph

**FIGURE 3:**
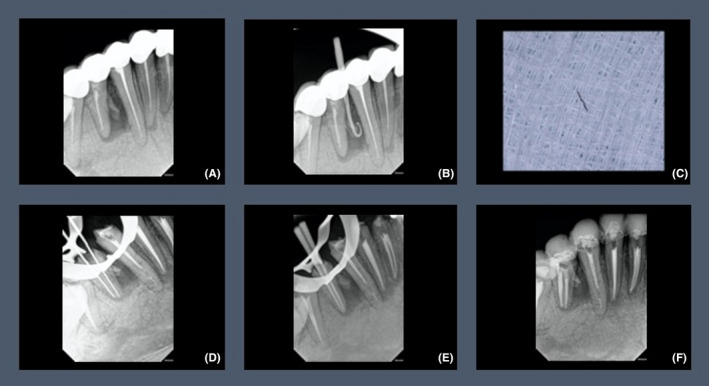
(A,B) Pre‐operative periapical radiograph; (C) fractured instrument removal; (D) working length radiograph; (E) master cone radiograph; (F) post‐operative radiograph

A 32‐year‐old fit and healthy Saudi male patient was referred from the prosthodontic department to the post‐graduate endodontic clinics at King Abdul‐Aziz University Dental Hospital (KAUDH), Jeddah, Saudi Arabia. Upon presentation, the patient complained from general discomfort upon eating along with a persistent pus discharge oozing frequently from the lower right area (Figure 1). Extra‐oral examination was normal, while intra‐oral examination revealed a sinus tract mesial to lower right premolar (#44), moderate tenderness to percussion, and palpation on tooth #44 and generalized mild gingival inflammation due to the ill‐fitted bridge fabricated several years ago. Periodontal examination showed deep pocket ranging from 6–7 mm mesial to #44. Then, cold test was done and showed negative response when compared to the neighboring first molar (#46) and the opposing first premolar (#14). Panoramic radiograph evaluation revealed a heavily restored dentition with multiple crowns. Several teeth were sub‐optimally root canal treated and were associated with periapical radiolucencies (Figure 2). Periapical (PA) radiograph was obtained for the offending tooth #44 and showed sub‐optimal root canal filling, and what appears to be a separated instrument inside the canal. Tooth #44 was also associated with an ill‐defined large radiolucency starting apically and extending almost to the middle of the root mesially. Adding to that, a radiopaque mass was found in close proximity to the same tooth (Figure 3A). Number 35 gutta‐percha point was used for tracing the sinus tract, which pointed to the foreign body (Figure 3B). A diagnosis of chronic apical periodontitis associated with an existing root canal filling and a draining sinus was reached for the lower first premolar tooth. A preliminary diagnosis for the foreign body was suggested to be either a retained remaining deciduous root, a lingually positioned additional root with an abrupt curvature or root fracture. After discussing various treatment options with the patient and as the patient was keen to try and save the tooth, it was decided to start with a non‐surgical root canal retreatment (NSRCRTx) and follow‐up on the symptoms of the patient. If symptoms were resolved, this will be followed by post, core, and full coverage restoration.

The patient was sent back to the prosthodontic department in order to remove the old bridge and replace it with a provisional one. Two weeks thereafter, the patient came back for his endodontic treatment. Initially, inferior alveolar nerve block (IANB) was achieved by administering 2% lidocaine with 1:80000 epinephrine (Dentsply Pharmaceutical); this was followed by removal of the temporary bridge and placement of rubber dam. Secondary caries was removed, and access cavity was modified. Two canals were identified, and D1, D2, and D3 ProTaper Retreat files (Dentsply Maillefer) were used sequentially to remove the old root canal filling. A broken instrument inside the canal was found between the gutta‐percha and the root canal wall in the buccal canal at the apical 4 mm. Missed lingual canal was detected under the microscope but was not negotiated until retrieval of the fractured instrument was done. The retrieval trial was attempted through trephination of dentin around the broken file in a counterclockwise direction utilizing Terauchi File Retrieval Kit ultrasonic tip 6 (TFRK‐6) attached to P5 Newtron ultrasonic machine (Acteon) set at low power of 4 KHz (Figure 3C). Working length (WL) was determined to be 15 mm for both canals using electronic apex locator (EAL) Root ZX II (J Morita, Tokyo, Japan) (Figure 3D). Further cleaning and shaping of both canals were done using OneCurve (Micromega) and X3 ProTaper NEXT (Dentsply Maillefer) NiTi instruments to the full WL. The canals were copiously irrigated with 5.5% sodium hypochlorite (NaOCl) using 27‐gauge needle 2 mm short of the working length throughout the treatment. Calcium hydroxide (CaOH_2_) (UltraCal XS, Ultradent Products Inc.) was placed into the canals followed by a temporary restoration.

In the second appointment, the patient was asymptomatic, but the sinus tract did not heal. Chemo mechanical preparation was continued; the canals were further irrigated with 17% ethylenediaminetetraacetic acid (EDTA) followed by a final flush with NaOCl. Master cones size #30/07 were inserted to the full WL and cemented with Zinc Oxide Eugenol (ZOE) sealer (Sealapex) (Sybron‐Endo) in a down‐pack with a continuous wave technique and back‐fill with gutta‐percha (Figure 3E,F). Finally, a temporary restoration was placed, and the patient was seen again after 1 month.

## 3 FOLLOW‐UP EXAMINATION

**FIGURE 4:**
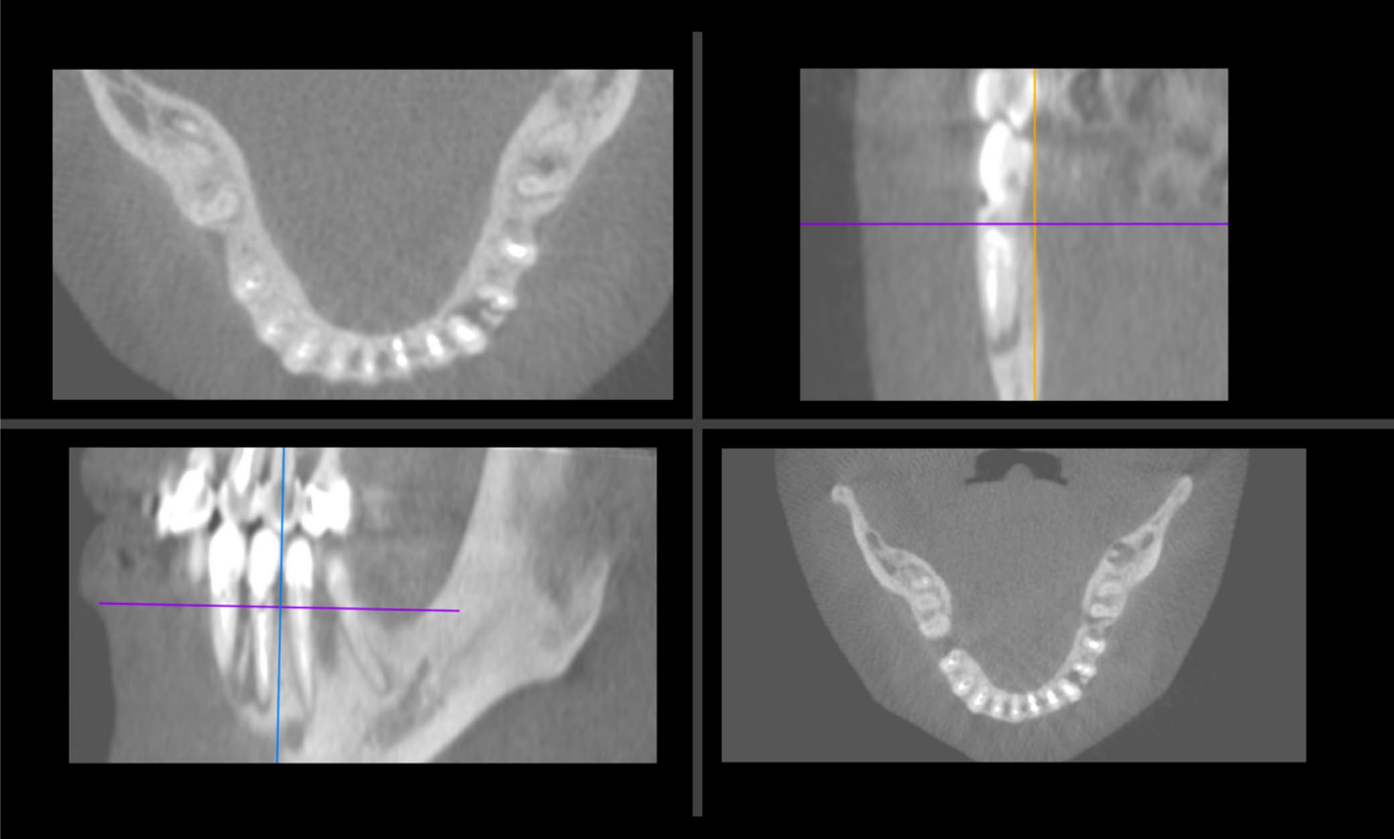
Cone‐beam computed tomography scan

**FIGURE 5:**
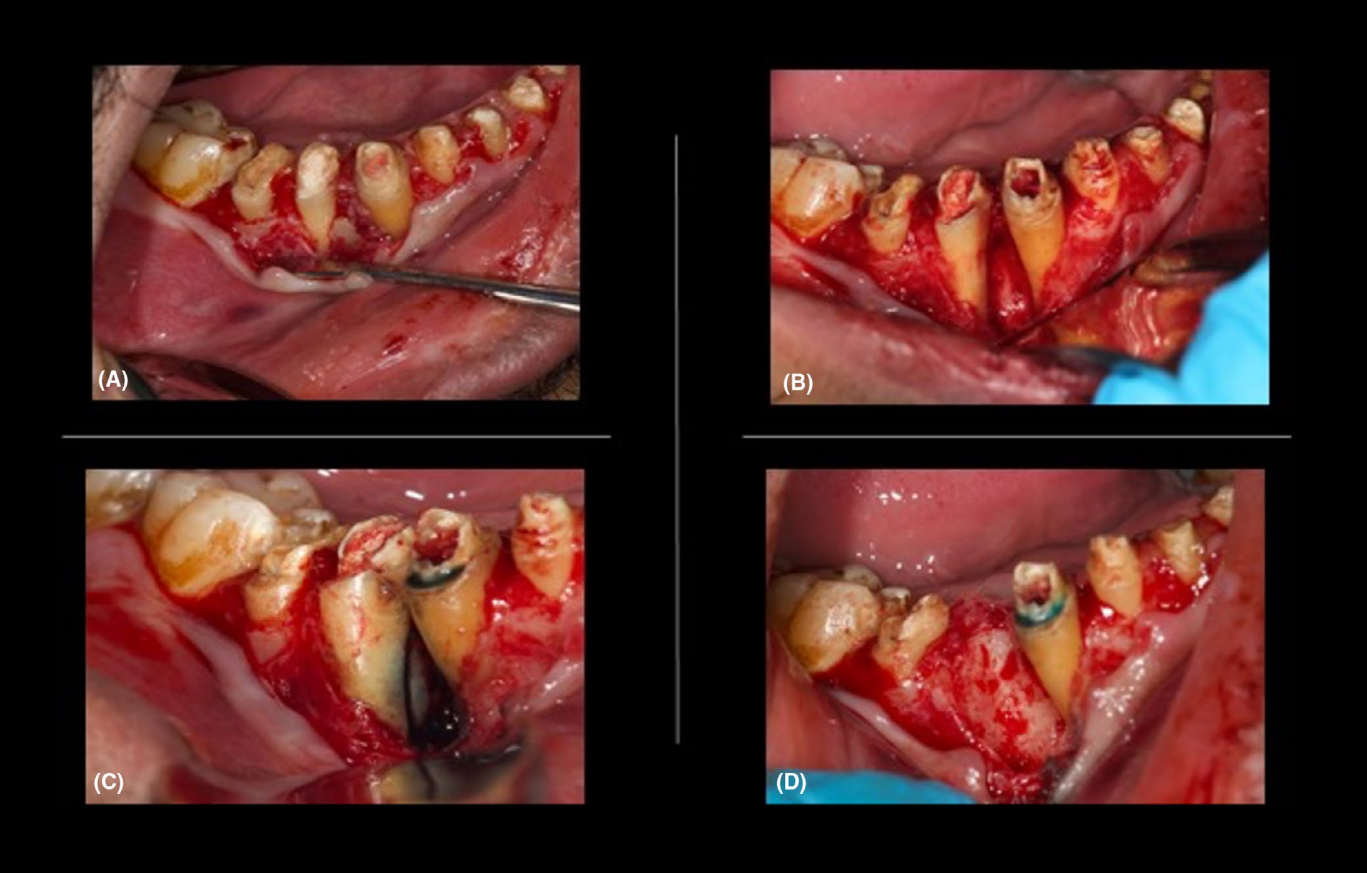
Intra‐operative images (A) retraction of flap showing granulation tissue (B) after removal of granulation tissue (C) toluidine blue stain showing VRF (D) after tooth extraction

One month after the treatment, the patient was seen and was asymptomatic, but the sinus tract did not heal. As the radio‐opaque foreign body was not identified as an extra‐root, it was decided to perform a CBCT scan for a pre‐surgical assessment of the lower right premolar tooth (Figure 4) and exploratory surgery to identify the radio‐opaque foreign body. Accordingly, the patient was referred to the department of periodontics for exploratory surgery. A scalloped sulcular incision using blade #15c extending one tooth interproximally with a full flap reflection was done. Eventually, the foreign body was found to be a piece of granulation tissue with bone sequestrum. A thorough scaling and root planning were done followed by application of a toluidine blue stain in a way to indicate a vertical root fracture (VRF). Unfortunately, it was obvious that a VRF extending along the whole length of the root on the mesial aspect was present (Figure 5). The final decision was made to have the tooth extracted and replaced with an implant after ridge augmentation within 6 weeks.

## 4 DISCUSSION

An effective root canal therapy can be accomplished only if all canals were identified, cleaned, shaped, filled to the full WL, and coronally sealed to prevent any further apical percolation of microorganisms.^24^ Cases with aberrant root canal morphology necessitate a contingency alteration in the original access preparation. Pre‐operative radiographs were not helpful in the present report, even though different angles were deemed at first. Regular conventional radiographs may fail to locate any additional root or canal. Hence, a limited field of view (FOV) CBCT which is lower in radiation dose would be a better alternative option in such cases with suspicious anatomy although it would be difficult to identify vertical root fractures from scans. Unsuccessful retreatment would be seen when ledges and broken files are found. Thus, debridement of the entire canal is nearly impossible.^25^, ^26^

To avoid all endodontic errors, six laws were suggested by Kranser and Rankow to help the clinicians in preventing such events although they were implemented for molars but such laws should be advocated for premolars.^27^ This would be easier when implemented with higher magnification and illumination. Breaking a file inside root canal system is an infelicitous event that may influence the treatment outcome.^28^ Two consequences have to be looked after once a broken file exists: First, corrosion which has limited support in the literature on its effect on the outcome and second obstructing the path reducing the chance of a thorough debridement. Strindberg pointed out a lowered healing rate by 19% when the file was left *in situ*.^29^ Another study with 2‐year follow‐up period exhibited a high success rate up to 89% for vital as well as necrotic cases under the condition of no apical radiolucencies. Otherwise, a massive reduction by 47% would result.^30^ Additional two outcome studies have shown no influence of broken files on outcome. The first study reviewed 8500 cases among which 168 were retaining files and showed a success rate of 81% when compared to controls.^31^ Furthermore, Spili et al. found similar results of 86.7% and 92.9% success rate in the separated instrument and the control groups, respectively.^32^ One of the highly recommended techniques in file retrieval is using ultrasonic trephination. Success rate of ultrasonic implementation in files retrieval was relatively high in the literature ranging between 88% and 95% resulting in better outcomes.^33^, ^34^

Vertical root fracture (VRF) is defined, according to the American Association of Endodontics (AAE), as “a longitudinally oriented fracture of the root that originates from the apex and propagates to the coronal part”.^35^ VRF is common in endodontically treated teeth, although it can occur in vital non‐restored teeth. It is considered to be the third most common cause for tooth loss in endodontically treated teeth with most susceptible teeth being premolars followed by molars, incisors, and canines.^36^, ^37^ Loss of tooth structure and loss of fracture resistance after endodontic preparation, root canal configuration, excessive forces during lateral condensation, as well as post type and materials all are predisposing factors for VRF.^38^ Clinical signs and symptoms vary according to extension and duration of the fracture. VRF may result in pain and abscess formation due to bacterial leakage and growth in the fracture space. In other situations, signs and symptoms might be confined to mild tenderness on mastication, dull discomfort or sinus tract close to the gingival margin, and isolated deep periodontal pocket.^39^ Early detection of this lesion may improve the prognosis tremendously; it prevents the unnecessary endodontic treatment and further periodontal destruction.

VRF is usually diagnosed by thorough clinical and radiographic examination. Previous studies showed that accurate detection of VRF can be spotted with CBCT slice thickness of 0 to 2 mm.^40^ Other methods of detection include application of dye solutions, indirect illumination of the root, fiber‐optic light, and tooth sloth. The diagnosis of a vertical root fracture can be confirmed by surgical exposure of the root for direct visual detection.^41^

## 5 CONCLUSION

Using advanced technologies like CBCT, magnification, and illumination is crucial for better diagnostic purposes and to avoid any procedural errors. Clinicians should also increase their knowledge on how to diagnose and manage VRF as this would prevent unnecessary treatment for patients.

## CONFLICT OF INTEREST

The authors deny any conflict of interest.

## AUTHOR CONTRIBUTIONS

*M. Yagmoor* involved in conceptualization, data curation, investigation, methodology, resources, visualization, writing–original draft preparation, and writing–review and editing. *A. Bakhsh* involved in conceptualization, data curation, methodology, project administration, visualization, and writing–review and editing. *O. Mandourah* involved in data curation, investigation, and visualization. *L. Alsofi* involved in methodology, project administration, supervision, and writing–review and editing.

## ETHICAL APPROVAL

None.

## CONSENT

Written informed consent was obtained from the patient to publish this report in accordance with the journal's patient consent policy.

## Data Availability

Research data are not shared.
